# Section 1. EPR-3 versus GINA 2008 Guidelines - Asthma Control and Step 3 Care: *Highlights of the Asthma Summit 2009: Beyond the Guidelines*

**DOI:** 10.1097/WOX.0b013e3181cb90c3

**Published:** 2010-02-15

**Authors:** Jean Bousquet, William W Busse

**Affiliations:** 1 Montpelier University and INSERM, Montpelier, France; 2 Department of Medicine, University of Wisconsin School of Medicine and Public Health, Madison, WI

**Keywords:** EPR-3, GINA, asthma, guidelines

## Abstract

Recent updates to asthma guidelines from the Global Initiative for Asthma (GINA) and the National Asthma Education and Prevention Program (Expert Panel Report 3, EPR-3) share many similarities, reflecting a focus on asthma control based on clinical manifestations of disease and responsiveness to therapy. Both documents build upon the recommendations of former guidelines utilizing evidence-based review of the published literature to revise algorithms for practice. A major difference between the 2 reports is the preferred treatment at Step 3. The GINA guidelines recommend a combination of low-dose inhaled corticosteroid (ICS) plus long-acting *β*-agonist (LABA), whereas the EPR-3 advises either monotherapy with medium-dose ICS or the low-dose ICS + LABA combination. Both approaches are supported by clinical experience and Level A evidence. The option of personalized therapy is a point of discussion for future guidelines.

## Introduction

The evolution of the Global Initiative for Asthma (GINA) guidelines reflects a shift in the paradigm for asthma treatment[1]. The 1995 and 2002 versions of the guidelines proposed that treatment decisions be based on disease severity[2,3]. The 1995 guidelines classified disease severity into one of 4 categories based on the frequency of symptoms, exacerbations, nocturnal symptoms, forced expiratory volume in the first second (FEV_1_), or peak expiratory flow (PEF) before treatment began[2]. However, many patients were already on treatment when physicians first saw them, making classification of disease severity difficult. Later discussion suggested accounting for these patients by classifying disease severity and recommending treatment based on clinical features and step of medication regimen[3]. However, this scheme was complex and presented a considerable challenge to primary care physicians, who are often the main providers for patients with asthma.

Data suggesting that poorly controlled asthma days were more important in treatment considerations[4] played an important role in providing the 2002 updated guidelines with a focus on asthma control[3]. The difficulty with this approach is that the definition of asthma control can differ based on the assessment used, and who is assessing control. For example, "control" may mean different things to the patient, the care-giver, the general practitioner, the respiratory physician, or to the regulatory authority. Furthermore, current treatment approaches are guided by single clinical end points, which might overestimate true asthma control. This was illustrated by a study showing that the percentage of patients exhibiting asthma control differed across single end points[5]. A composite measure of control might help to improve outcomes.

**Figure 1:**
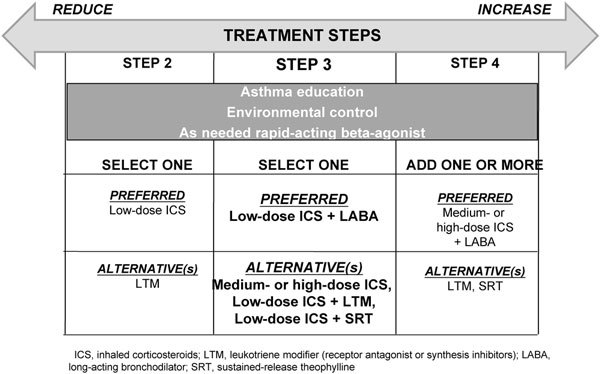
**GINA stepwise approach for managing asthma in patients ≥ 5 years of age with persistent mild or moderate disease, focus on step 3 **[1].

The 2008 GINA guidelines define severity based on clinical manifestations of the disease and how these manifestations respond to therapy (Table 1)[1]. However, in recognition that asthma severity can change over time, the guidelines no longer recommend classification of asthma severity for treatment decisions. Instead, GINA 2008 classifies asthma by level of control and uses a protocol of 5 treatment steps as shown in Figure 1; the former severity-based classification is recommended only for research purposes[1]. In this manner the most recent GINA guidelines recommend that treatment decisions be made based on the patient's level of asthma control.

**Table 1:** 2008 GINA Guidelines Emphasize Asthma Control[1]

Characteristic	Controlled	Partially Controlled	Uncontrolled
Daytime symptoms	None (twice or less per week)	More than twice per week	Three or more features of partially controlled asthma in any week
Limitations of activities	None	Any	
Nocturnal symptoms	None	Any	
Need for reliever rescue medication	None (twice or less per week)	More than twice per week	
Lung function (FEV_1 _or PEFR)	Normal or near-normal	Less than 80% predicted or personal best	
Exacerbations	None	One or more per year, although any exacerbation should prompt review of maintenance treatment to ensure it is adequate	One in any week
Treatment	Maintain treatment and find lowest controlling step	Consider stepping up to gain control, and treat exacerbations	Step up treatment until disease is controlled, and treat exacerbations

According to GINA 2008, the preferred treatment for Step 3 (ie, for patients with moderate, persistent asthma) is a combination of low-dose inhaled corticosteroids (ICS) and long-acting *β*-agonist (LABA) for adults and medium-dose ICS for children[1]. This is based on a rich body of data from randomized, controlled trials. In particular, clinical experience has supported the findings of the budenoside/formoterol (Symbicort) Maintenance and Reliever Therapy (SMART) study, which showed that using an ICS/LABA combination can effectively reduce exacerbations and improve overall asthma control[6]. Because of the evidence linking LABA use alone with increased risk for asthma-related death, GINA 2008 emphasizes that LABA should not be used regularly as monotherapy, and the document no longer lists LABA alone as an option for add-on therapy at any step[1]. Optional treatments for step 3, including increasing the dose of ICS (particularly, in children) or using a combination of ICS with leukotriene receptor antagonist or theophylline. Having options such as these enhances the flexibility of treatment to better meet patient needs.

There are more similarities to GINA 2008 and guidelines from the National Asthma Education and Prevention Program Expert Panel Report 3 (EPR-3) than differences[1,7]. Both define asthma control based on clinical manifestations and their responsiveness to therapy; both use similar language with respect to exacerbations; and both propose similar steps in asthma management, including the partnership between patient and physician. The differences that exist are slight: for example, GINA 2008 includes response to therapy in assessments of asthma control, whereas EPR-3 addresses resistance to treatment and effect of comorbid conditions. In addition, the EPR-3 assessment of asthma control is divided into domains of impairment and risk, which are described more fully in Dr. Busse's article after this. The impairment domain is very similar to the GINA control categories shown in Table 1.

In summary, many of the differences between the most recent GINA and EPR-3 guidelines are semantic in nature. These guidelines are excellent resources for specialists, but probably not yet appropriate for the majority of general practitioners who daily treat patients with asthma. We need to now give consideration to developing a single, simplified, and truly global guideline for those practitioners who see most of the asthma patients worldwide.

## A Choice At Step 3: The Naepp EPR-3 Guidelines

The National Heart, Lung, and Blood Institute (NHLBI) of the National Institutes of Health released in 2007 an update of the U.S. asthma clinical practice guidelines, EPR-3: Guidelines for the Diagnosis and Management of Asthma. This 400+ page document incorporates an evidence-based assessment of former guidelines and current literature in revising the recommendations for practice[7]. The complete guidelines are available at http://www.nhlbi.nih.gov/guidelines/asthma/asthgdln.htm.

**Figure 2:**
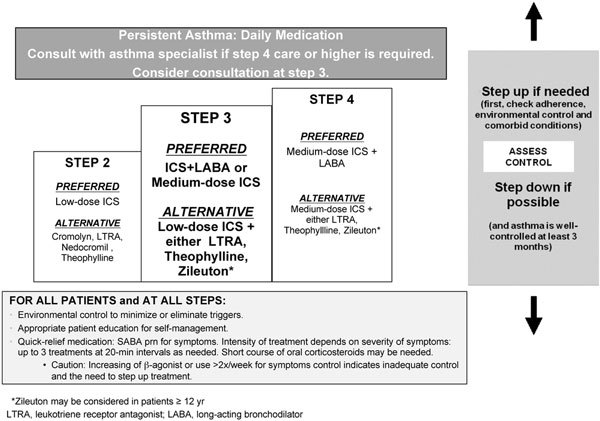
**EPR-3 stepwise approach for managing asthma in patients ≥ 5 years of age with persistent mild or moderate disease, focus on step 3 (adapted charts for children 5-11 years of age and youths ≥ 12 years of age and adults) **[7].

The major difference between the 2007 EPR-3 and earlier versions of the US guidelines is a focus on asthma control, which is defined as the degree to which manifestations of asthma (ie, symptoms, functional impairments, risk of untoward events) are minimized and the goals of therapy are met[7]. Asthma control is differentiated from asthma severity, which is defined as the intrinsic intensity of the disease process, and which is most accurately determined before a patient is placed on long-term therapy[7]. Classifying asthma severity is done to initiate treatment; assessing asthma control is done for ongoing monitoring; and both are broken down to domains of impairment and risk to operationalize therapeutic targets as described in Table 2. The therapeutic targets are defined by a step paradigm (Figure 2), and when asthma is well controlled at any level, improvements are observed in both domains [7].

**Table 2:** Criteria for Targeting the Domains of Asthma Control According to the EPR-3: Reducing Impairment and Risk[7]

Asthma Control Domain	Criteria
Reducing impairment	• Prevent chronic and troublesome symptoms (eg, coughing or breathlessness in the daytime, nighttime, after exertion)
	• Infrequent use (£2 days/week) of inhaled short-acting bronchodilator for quick relief of symptoms
	• Maintain (near) "normal" pulmonary function
	• Maintain normal activity levels (including exercise, other physical activity, attendance at work or school)
	• Meet patients' and families' expectations of and satisfaction with asthma care
Reducing risk	• Prevent recurrent exacerbations of asthma; minimize emergency department visits, urgent care visits, hospitalizations
	• Prevent progressive loss of lung function (for children, prevent reduced lung growth)
	• Provide optimal pharmacotherapy with minimal (ideally, no) adverse effects

For the most part, differences between the EPR3 and the 2008 GINA guidelines are indeed a matter of semantics. However, as shown in Figures 1 and 2, the 2 guidelines differ in what they recommend as preferred treatment at Step 3[1]. The GINA guidelines recommend a combination of low-dose inhaled corticosteroid (ICS) and long-acting *β*-agonist (LABA) as the preferred treatment,[1] whereas the EPR-3 recommends a more flexible approach that includes the option of either combination therapy or increasing the dose of ICS[1]. This is a greater degree of flexibility than earlier US guidelines, and it is based on Level A Evidence [7-10].

This change in the US guidelines has not been without controversy. LABAs have been shown to improve lung function, decrease asthma symptoms and exacerbations, and lower the ICS dose needed for control. However, the SMART reported an increased risk of asthma-related mortality,[11] and higher doses of formoterol have been associated with an increased likelihood of severe asthma exacerbations[12]. Thus, all agents that include a LABA must carry a Black Box warning in the US; and the EPR-3 recommends that potential benefits of using LABAs daily be balanced against the suggested risks [7].

It should be noted that these serious adverse events were reported in patients who largely used LABAs alone[11,12]. Furthermore, SMART was conducted in patients with severe disease[11]. Results from recent studies have found no increased risk for death when LABA are used in combination with ICS[13]. In fact, the number of asthma-related deaths have decreased with the advent of ICS/LABA combinations.

Several studies have suggested that adding a LABA is more effective than increasing ICS dose for improving impairment[14,15]. For example, in the Gaining Optimal Control of Asthma (GOAL) study, among patients with more severe asthma, the percentage of those achieving control was higher for patients taking a fluticasone propionate/salmeterol combination than for those taking fluticasone alone[8]. Likewise, in the Formoterol and Corticosteroids Establishing Therapy (FACET) study, a combination of low-dose budesonide and formoterol appeared to be more effective than using budesonide alone [16].

The distinction in terms of clinical benefit between increasing the ICS dose and using an ICS/LABA combination at Step 3 is not always clear cut. For example, in the GOAL study, the percentage of patients with less severe disease achieving well-controlled asthma was similar between those using an increased dose of fluticasone alone and those given the fluticasone/salmeterol combination[8]. In the FACET study, patients using increased doses of budesonide monotherapy had fewer severe exacerbations than patients using the budesonide/formoterol combination[16]. However, another study showed that the budesonide/formoterol combination delayed severe exacerbations when it was used both as maintenance and reliever therapy, in contrast to either increasing the dose of budesonide monotherapy or using the combination as maintenance with a short-acting *β*-agonist (SABA) as reliever[17]. In this study the number of severe exacerbations was comparable for the increased ICS and ICS/LABA + SABA treatments.

In a recent Canadian registry study the proportion of patients receiving successful treatment (defined as no hospitalizations, no use of oral corticosteroids, and use of less than 1 dose per day of SABA) was compared with the effectiveness of either increasing the current dose of ICS or adding a LABA[18]. Both approaches were similar when the comparative outcome was symptoms, that is, impairment. In contrast, when the need for frequency of oral prednisone courses was evaluated, those who received an increase in ICS had a reduced need for prednisone bursts. An accompanying editorial suggested that increasing the ICS dose permits better control of inflammation, making the airways less susceptible to exacerbation, whereas adding a LABA most likely reduces measures of impairment[19]. This raised the question that the addition of a LABA reduced symptoms but not underlying inflammation. With the additional ICS, there was a reduction in airway inflammation and with this effect, fewer exacerbations. In addition, we should consider whether there is a group of patients for whom combination therapy does not work, or for whom monotherapy works better. Biomarkers are needed to identify those patients. Sputum eosinophils might be useful in signaling propensity for exacerbation, but their use as a marker is still limited[20]. Patient preferences also must be considered. Interestingly, most patients are not concerned so much with exacerbations as they are with risks for side effects. Thus, the debate about increased ICS dose versus combination therapy with a LABA is likely to continue for some time.

Several questions remain with respect to comparisons between the EPR-3 and GINA guidelines. For example, the best approach for stepping down treatment is not clear; both guidelines address this issue, but data are limited[1,7]. In addition, more study is needed in children. Most of the data supporting increased ICS dosage or addition of a LABA come from adult studies. As stated in the EPR-3 guidelines, there are not enough data to substantiate a benefit for LABA use in children younger than 11 years[7]. In addition, it is not clear how best to approach exacerbations that show no eosinophil infiltration and are, thus, unresponsive to corticosteroids. The mechanism underlying these exacerbations is not clear, and it could be that different triggers dictate different pathways to exacerbations.

## Section 1 Discussion

Dr. Bousquet: I have a few comments. I think the concept of impairment and risk is very good and may provide a better approach to treatment. Guidelines are evolving. However, I believe that there should be a common guideline, a common approach. In fact, the various guidelines are not very different. Also, in terms of treatment what is important in today's guidelines are combination therapy with an ICS and LABA in a single inhaler and also using ICS and then doubling the dose when there is an exacerbation.

Dr. Busse: I would agree.

Dr. Bousquet: The guidelines are moving us toward a flexible approach to therapy, even in the States. My usual treatment is the SMART approach, that is to say flexible therapy. Clearly, it reduces impairment; the number of serious exacerbations goes down. I don't know if it substantially reduces risk. The problem is to me compliance. Most of the patients I see with severe asthma are patients who do not take their treatment.

Dr. Busse: I think what we'd like to have is some marker we could use to find out who's at risk. At this time there are a limited number of options. Exhaled nitric oxide certainly is a marker of airway inflammation and may be helpful in knowing when to reduce ICS, but it may not necessarily tell us when we have to up-regulate the dose. Sputum eosinophils mark a propensity toward an exacerbation, but most places have limitations in being able to do this.

Dr. Oppenheimer: Patient preference should be a factor when assessing risk. Apparently, some patients are not as concerned about the risk of an exacerbation as they are about the risk of sudden death with LABA or about side effects of ICS; and they make their decision on what they'll comply with often on those aspects.

Dr. Bousquet: Frankly speaking, this is more of a US concern. However, steroid phobia is a major concern in France, which is why I use more combination therapy than ICS monotherapy.

Dr. Busse: I think the concerns about LABAs are more of a scare than reality. But, how you can quell your patients when they hear this in the news?

Dr. Storms: I have a question about the risk domain. Let me use a case scenario. A patient comes in on combination therapy in June and gives a history of having had 2 prednisone bursts in the winter due to URIs. The rest of the year her asthma is totally controlled. Pulmonary function is 85% predicted FEV_1_. Based on the risk domain, what would you tell her?

Dr. Busse: That's a good question. Based on the GOAL data, the exacerbations should decrease by either increasing the dose of ICS or ICS + LABA in combination. But, more importantly, the longer you use the higher doses of either approach, the lower the rate goes. For some patients who are "exacerbation prone," increasing the dose and maintaining the increase over a longer period of time might protect them from an exacerbation to some degree. It's not perfect, though.

Dr. Storms: So you would increase the dose all year?

Dr. Busse: Yes, absolutely.

Dr. Bousquet: I might do something else. I would increase the dose of combination therapy as the patient has a cold.

Dr. Gelfand: It seems to me that the triggers of the exacerbation dictate the pathways that are activated and, ultimately, the therapeutic arms that are going to be effective. Many exacerbations, depending on the trigger, have no eosinophils and are unresponsive to corticosteroids.

Dr. Bousquet: I cannot agree more; but the data consistently have shown that if you increase the dose of combination therapy early on in the exacerbation, you reduce the risk of exacerbation and subsequent exacerbations.

Dr. Busse: I agree that the mechanism by which these people exacerbate is important, and until we understand the mechanism, we don't know what we're going after. I think it's an unmet need.

Dr. Hargreave: We've heard about stepping up to bring about control, but not about then stepping down. And this can have different consequences. For example, in stepping up control, as we've heard, you might use combination therapy. But, once control is achieved, combination therapy may no longer be needed, or the dose of ICS might need to be reduced. Can you comment on that?

Dr. Bousquet: Well, in the GINA guidelines, there are only B and D evidence for stepping down.

Dr. Busse: The EPR-3 didn't address it either due to the lack of data. A marker of inflammation would be helpful, because right now it's arbitrary--if you're stable for 3 months, consider a reduction; yet, 3 months may not be sufficient time to stabilize. It's an area that hasn't been addressed very well.

Dr. Bukstein: Most of the people instituting guidelines today are primary care physicians (PCPs), and they have multiple guidelines. One of the things that most of their guidelines stress, which PCPs are used to, is patient lifestyle. We haven't talked about lifestyle change.

Dr. Spector: That is a good point. In the real world people have their own ideas, and there can be some big differences ethnically. We just completed a survey of patients and PCPs about guidelines and controller medications. More than 25% of the patients and 14% of the physicians stated that they would stop the medication when asymptomatic. The percentages are greater in Blacks and in Hispanics who have different concepts of care and, maybe, different access to care.

Dr. Busse: Let me comment a little further. In an asthma control evaluation conducted in inner cities in the US in a high-risk group of patients, we looked at whether care would improve if a biomarker, in this case exhaled nitric oxide (eNO), was added to a guideline-based algorithm for treatment. Under those circumstances, the additional marker of inflammation wasn't very helpful. During the study adherence with care was high, but when the study ended, many patients went back to previous ways of managing their disease. They didn't have medication available. So, it's not just the guidelines. It's having a way of implementing and applying them that make the difference. Some of this may be social and some of it may be economic, but both are important.

Dr. Brightling: Related to this, the observation that almost without exception patients get better in clinical trials has actually been the undoing of a lot of studies because it obviously impacts on the exacerbation frequency during the study. Clearly, it's partly due to improvements in care, access to care, and access to treatment; but it's also related to how patients adapt to actually tolerate their poor disease control. For example, I saw a patient who is in his 30s in our difficult asthma clinic He usually cycles to work, but told me that he had stopped for the last 6 months due to poor asthma control. Instead of increasing his therapy, he tolerated the fact that he could not cycle and had to drive. Many patients dislike their therapy; and they actually adapt their lifestyles around being able to tolerate poor control.

Dr. Oppenheimer: I would agree. I think there are 2 domains of impediment. The first is the doctor, and the second is the patient. The asthma guideline is estimated to have been read by about 75% of PCPs, but the busiest clinicians tended to use it the least. Yet, these are the very people who we are writing these guidelines for; they take care of the majority of asthmatics. We keep talking about spirometry being a useful tool and the fact that there's no one measure of control. Are PCPs really going to adhere to the guidelines if they are not doing spirometry, and how much are we really missing?

Dr. Bukstein: It's not just physicians and patients; it's also the regulatory bodies. One of the problems with guidelines is that they are used by people who regulate how we practice, converting them into a step care plan that fits their paradigm of decreasing costs.

As far as lung function testing, even if we could get PCPs to do lung function testing, I doubt that it would result in a lot of change. I think what is more important is an emphasis on unifying the risk benefit analysis for the physician and the patient, bringing them closer together, understanding the burden of disease and the burden of therapy to assess potential benefit.

Dr. Brightling: In the UK the uptake for spirometry by PCPs has increased enormously over the last 2 years because they are now funded to do regular spirometry on patients with chronic lung disease. This is ironic. As Dr. Bousquet pointed out an important difference between the EPR-3 and GINA guidelines is the value of doing repeated measures of spirometry within a primary care population. It has value in terms of trying to establish a diagnosis, but in the EPR-3 guidelines spirometry also has a major role in terms of looking at risk. I would challenge that view and suggest that it may be overstated. The level of spirometry may not be as closely related to risk of exacerbations as we think. Other control measures, such as the ACQ 6 may be more valuable. Clearly, if you look at the difficult asthmatics, there is a population of patients who have unstable disease and frequent exacerbations, but normal spirometry between episodes.

Dr. Bousquet: Spirometry in GP offices may not be very reliable; it's not so easy to do, it's more complicated than doing blood pressure.

Dr. Busse: I think that spirometry is a measure by which you can look at one end point, but there's variability. However, as Dr. Bousquet indicated, many times people don't perceive their fall in lung function because it's so gradual. They can be living at 60% and accept it, and they adjust their lifestyle rather than do anything. It underscores the fact that asthma in many cases is a very complex disease, which requires sophistication in terms of evaluation and understanding of the disease.

Dr. Bukstein: But shouldn't the guidelines take a more practical approach? Maybe there should be separate guidelines for PCPs taking into account what they can and cannot do on a practical basis. Also, should guidelines address things outside of pharmacologic therapy in a more aggressive manner, things like how drugs are paid for and the importance of access to medication, to treatment? Again, one of the most common reasons that exacerbations occur is poor access to medications.

Dr. Bousquet: One problem is that guidelines are usually written by the most sophisticated doctors who see the most severe patients. Patients and GPs are not included. This needs to be fixed because if you write guidelines which are not applicable in primary care, they will not be used. Also, if you write guidelines that are not agreeable to patients, they will not be used.

Another issue is to keep things simple. The concept of risk and benefit makes things more complex. So, is this an approach for the specialist or is it an approach for the GP?

Dr. Busse: We need to recognize that many patients do read the guidelines, and they emerge with an understanding of what expectation should be as far as their disease control. The NAEPP did point out times when it is important to see the consultant, particularly for patients with frequent exacerbations. Cost of medications is political, but patient expectations for disease control may be very helpful.

Dr. Oppenheimer: What about alternative dosing?

Dr. Busse: To some extent this is FDA driven. But, I agree that we need to start to make adaptations to the phenotype of the patient. Some patients can do very well on episodic treatment; others might do well on other forms of treatment. We need to be innovative, not rigid. The guidelines are a framework, not rules.

Dr. Spector: Some patients cannot afford ICS or combination therapy. Do we need to have other guidelines for patients who can't afford the medication that we're advocating?

Dr. Bousquet: As many as 50% of the world population cannot get access to ICS; yet, this is the critical drug. ICS can reduce most of the severity of asthma and most of the exacerbations. So, we need to make available generic, low-cost, ICS of high quality.

Dr. Oppenheimer: There's a lot of interesting data coming out on anxiety and depression having significant impact on patient perception about disease control. Is that a domain that we're not addressing aggressively enough?

Dr. Bukstein: Depression, and all the other parts of lifestyle, is incredibly important.

Patients need to have better self efficacy if we're going to help them control their disease, and one thing that helps with self efficacy is the idea that the individual can modify his or her therapy, that they are empowered. Flexibility, however it is built it into a patient's therapy, is going to be very helpful.

Dr. Busse: An interesting study in a mouse model showed that a chronic increase in anxiety was associated with steroid insensitivity. It makes you wonder about the brain connection. The depressive index in asthma is extremely high. How that integrates with responsiveness to therapy may be important.

Dr. Calhoun: So, how can we put some of this into action? Could we have our medical societies work with vendors of electronic medical records (EMR) to implement guidelines? The EMR is clearly the wave of the future.

Dr. Bukstein: I think that's key. System change will yield the biggest benefit, but I think it would take a real push between all the organizations to do that. The only way we're going to get PCPs to really institute guidelines is to model that with some sort of interactive information. As the guidelines evolve, some thought has to be given as to how the information is going to be transmitted to primary care.

## Note

Research support from National Institutes of Health, Novartis, Centocor, GlaxoSmithKline, MedImmune, Ception.

Jean Bousquet, MD, is consultant for ALK, AstraZeneca, Sanofi-Aventis, Chiesi, Fabre, GlaxoSmithKline, Lehti, Merck, Sharp & Dohme, Novartis, Schering Plough, Stallergenes; Speakers' Bureau - Novartis, Stallergenes; William Busse, MD, is on the advisory boards of Altair, Merck, Wyeth, Pfizer, Centocor, Amgen, UCB, Johnson & Johnson, and Bristol-Meyers Squibb; consultant for Novartis, AstraZenea, Eisai, TEVA, CompleWare, KaloBios, Boehringer Ingelheim, and Sandoz.

## References

[B1] Global Initiative for Asthma (GINA) Global strategy for asthma management and prevention 2008 http://www.ginasthma.org Accessed December 2008

[B2] Global Initiative for Asthma (GINA) Global strategy for asthma management and prevention. NHLBI/WHO Workshop report 1995 Bethesda, MD: National Institutes of Health Publication No. 95-3659

[B3] Global Initiative for Asthma (GINA) Global strategy for asthma management and prevention. NHLBI/WHO Workshop report Bethesda, MD: National Institutes of Health Publication No. 95-3659 updated 2002. Available at: http://www.ginaasthma.com. Accessed December 2008

[B4] O'Byrne PM Barnes PJ Rodriguez-Roisin R Runnerstrom E Sandstrom T Svensson K Tattersfield A Low dose inhaled budesonide and formoterol in mild persistent asthma: the OPTIMA randomized trial Am J Respir Crit Care Med 2001 3 1392 1397 10.1164/ajrccm.164.8.2104102 11704584

[B5] Irvine L Crombie IK Alder EM Neville RG Clark RA What predicts poor collection of medication among children with asthma? A case-control study Eur Respir J 2002 3 1464 1469 10.1183/09031936.02.00302102 12503705

[B6] Kuna P Peters MJ Manjra AI Jorup C Naya IP Martinez-Jimenez NE Buhl R Effect of budesonide/formoterol maintenance and reliever therapy on asthma exacerbations Int J Clin Pract 2007 3 725 736 10.1111/j.1742-1241.2007.01338.x 17362472 PMC1920547

[B7] National Asthma Education and Prevention Program Expert Panel Report 3. Guidelines for the diagnosis and management of asthma 2007 National Heart, Lung, and Blood Institute http://www.nhlbi.nih.gov/guidelines/asthma/10.1016/j.jaci.2007.09.043 17983880

[B8] Bateman ED Boushey HA Bousquet J Busse WW Clark TJ Pauwels RA Pedersen SE GOAL Investigators Group Can guideline-defined asthma control be achieved? The Gaining Optimal Asthma ControL study Am J Respir Crit Care Med 2004 3 836 844 10.1164/rccm.200401-033OC 15256389

[B9] Greenstone IR Ni Chroinin MN Masse V Danish A Magdalinos H Zhang X Ducharme FM Combination of inhaled long-acting beta2-agonists and inhaled steroids versus higher dose of inhaled steroids in children and adults with persistent asthma Cochrane Database Syst Rev 2005 3 CD005533 16235409 10.1002/14651858.CD005533

[B10] Masoli M Weatherall M Holt S Beasley R Moderate dose inhaled corticosteroids plus salmeterol versus higher doses of inhaled corticosteroids in symptomatic asthma Thorax 2005 3 730 734 10.1136/thx.2004.039180 16135679 PMC1747519

[B11] Nelson HS Weiss ST Bleecker ER Yancey SW Dorinsky PM The Salmeterol Multicenter Asthma Research Trial: a comparison of usual pharmacotherapy for asthma or usual pharmacotherapy plus salmeterol Chest 2006 3 15 26 10.1378/chest.129.1.15 16424409

[B12] Mann M Chowdhury B Sullivan E Nicklas R Anthracite R Meyer RJ Serious asthma exacerbations in asthmatics treated with high-dose formoterol Chest 2003 3 70 74 10.1378/chest.124.1.70 12853504

[B13] Nelson HS Long-acting beta-agonists in adult asthma: evidence that these drugs are safe Prim Care Respir J 2006 3 271 277 10.1016/j.pcrj.2006.08.006 16979380 PMC6730834

[B14] Woolcock A Lundback B Ringdal N Jacques LA Comparison of addition of salmeterol to inhaled steroids with doubling of the dose of inhaled steroids Am J Respir Crit Care Med 1996 3 1481 1488 10.1164/ajrccm.153.5.8630590 8630590

[B15] Greening AP Ind PW Northfield M Shaw G Added salmeterol versus higher-dose corticosteroid in asthma patients with symptoms on existing inhaled corticosteroid. Allen & Hanburys Limited UK Study Group Lancet 1994 3 219 224 10.1016/S0140-6736(94)92996-3 7913155

[B16] Pauwels RA Lofdahl CG Postma DS Tattersfield AE O'Byrne P Barnes PJ Ullman A Effect of inhaled formoterol and budesonide on exacerbations of asthma. Formoterol and Corticosteroids Establishing Therapy (FACET) International Study Group N Engl J Med 1997 3 1405 1411 10.1056/NEJM199711133372001 9358137

[B17] O'Byrne PM Bisgaard H Godard PP Pistolesi M Palmqvist M Zhu Y Ekstrom T Bateman ED Budesonide/formoterol combination therapy as both maintenance and reliever medication in asthma Am J Respir Crit Care Med 2005 3 129 136 10.1164/rccm.200407-884OC 15502112

[B18] Thomas M von Zeigenweidt J Lee AJ Price D High-dose inhaled corticosteroids versus add-on long-acting beta-agonists in asthma: an observational study J Allergy Clin Immunol 2009 3 116 121 10.1016/j.jaci.2008.09.035 18986690

[B19] Sears MR Step-up therapy in uncontrolled asthma: choices and outcomes J Allergy Clin Immunol 2009 3 122 123 10.1016/j.jaci.2008.10.045 19062084

[B20] Green RH Brightling CE McKenna S Hargadon B Parker D Bradding P Wardlaw AJ Pavord ID Asthma exacerbations and sputum eosinophil counts: a randomized controlled trial Lancet 2002 3 1715 1721 10.1016/S0140-6736(02)11679-5 12480423

